# Long Term Glaucoma Drug Delivery Using a Topically Retained Gel/Microsphere Eye Drop

**DOI:** 10.1038/s41598-017-09379-8

**Published:** 2017-08-17

**Authors:** Morgan V. Fedorchak, Ian P. Conner, Joel S. Schuman, Anthony Cugini, Steven R. Little

**Affiliations:** 1 UPMC Eye Center, Department of Ophthalmology, University of Pittsburgh School of Medicine, Pittsburgh, USA; 2 The Louis J. Fox Center for Vision Restoration, University of Pittsburgh, Pittsburgh, PA USA; 3 Department of Chemical Engineering, University of Pittsburgh, Pittsburgh, PA USA; 4 Department of Clinical and Translational Science, University of Pittsburgh, Pittsburgh, PA USA; 5 Department of Bioengineering, University of Pittsburgh, Pittsburgh, PA USA; 6 Department of Immunology, University of Pittsburgh, Pittsburgh, PA USA; 7 The McGowan Institute for Regenerative Medicine, University of Pittsburgh, Pittsburgh, PA USA; 8 Department of Ophthalmology, Langone Eye Center, NYU School of Medicine, New York, NY USA; 9 Department of Neuroscience, NYU School of Medicine, New York, NY USA; 10 Department of Physiology, NYU School of Medicine, New York, NY USA; 11 Depatment of Electrical and Computer Engineering, NYU Tandon School of Engineering, New York, NY USA

## Abstract

The purpose of this study was to characterize and determine the efficacy of a long-term, non-invasive gel/microsphere (GMS) eye drop for glaucoma. This novel drug delivery system is comprised of a thermoresponsive hydrogel carrier and drug-loaded polymer microspheres. *In vitro* release of brimonidine from the GMS drops and gel properties were quantified. A single brimonidine-loaded GMS drop was administered to 5 normotensive rabbits and intraocular pressure (IOP) was monitored for 28 days. Here we report that IOP reduction in rabbits receiving a single brimonidine GMS drop was comparable to that of rabbits receiving twice daily, standard brimonidine drops. GMS drops were retained in the inferior fornix in all animals for the length of the study. Our results suggest *in vivo* efficacy over 28 days from a single GMS drop and a potential decrease in systemic absorption, based on a lack of substantial IOP effects on the fellow untreated eye, compared to brimonidine twice-daily eye drops. To our knowledge, this represents the first long-term, drug-releasing depot that can be administered as a traditional eye drop.

## Introduction

Glaucoma is the second leading cause of blindness worldwide and is associated with high intraocular pressure (IOP) as a risk factor^1^. Further, the overall prevalence of glaucoma in the US expected to nearly triple by the year 2050^2^. The most common glaucoma treatment is self-administration of topical, ocular hypotensive eye drop medication^3^. Failure to administer intraocular pressure (IOP)-reducing eye drops on the appropriate schedule can lead to progression of glaucoma, with consequent vision loss and blindness. Increased morbidity due to glaucoma progression results in significantly increased healthcare costs^4^. As low as approximately 30% of glaucoma patients are reported to have high levels of adherence to topical eye drop treatment^5, 6^. In the majority of patients, adherence rates are consistently below 70%^7^, largely due to the required frequency of drop administration^8^ and to a lesser extent difficulty of drop administration^9^.

Failure to adhere to prescribed treatment leads to a greater number of and more frequent office visits for patients, with consequent increases in cost to the medical system. Further, it leads to more medications being added to the patient’s medical regimen, as the physician is led to believe that the drops prescribed are not working, when in fact they are just not getting into the patient’s eye. In the end, non-adherence to medical therapy results in increased blindness in the population due to inadequately controlled disease, and more surgery, with associated complications, than would otherwise have to be performed.

Unfortunately, even correct drop administration remains problematic, with less than 10% of the total amount of drug taken up into the treated tissues^10^. The remainder of the drug is lost via overflow or is taken up systemically through the nasolacrimal drainage system^11, 12^. This inefficiency can lead to systemic side effects and necessitates high levels of drug per drop to achieve therapeutic effects. For this reason, a wide variety of alternatives to traditional eye drops for glaucoma are currently being explored, including but not limited to contact lenses, ocular inserts, injections, and various additives to topical drops^13–16^.

Attempts to increase efficiency and adherence rates for glaucoma medication have seen success with sustained release formulations. Systems reported to date typically require injection, for example into the punctum^17, 18^, (including for lacrimal occlusion in conjunction with topical medication)^19^, subconjunctival space^20–22^, suprachoroidal space^23^, or anterior chamber^24^. One such study demonstrated release of IOP-lowering drug *in vivo* for up to 120 days from a subconjunctival depot^20^. Additionally, the total loading of drug in such systems is much lower than for conventional eye drops, as with IOP reduction accomplished in our subconjunctival injection model using approximately 100 times less drug than twice-daily drops^21^. Despite these advantages, approximately 40% of patients are reluctant to receive regular subconjunctival injections^25^. Intraocular injections that may last longer would similarly require clinician administration and carry risks such as inflammation, infection, retinal detachment, and hemorrhage^26^. Further, intraocular injections are contraindicated for certain patients^27^ and retention of punctal plugs has been problematic^28^.

We hypothesized that slowly dissolvable/degradable materials such as poly(lactic-co-glycolic acid) microspheres^29^ could provide the necessary long term release in a topical eye drop-like formulation if there were a way to combine these microspheres with an appropriate carrier and retention material. To that end, thermoresponsive hydrogels are ideal as a matrix for drug-loaded microspheres that would be delivered topically, as they undergo a reversible phase transition from liquid to gel upon increasing temperature^30^. A reverse thermal, acrylamide-based hydrogel was tuned to have the desired physical properties, including rigidity and opacity, to serve as a sustained release eye drop material. Together, the thermoresponsive hydrogel carrier loaded with rate controlling, safe, and biodegradable polymer microspheres (henceforth referred to as gel microsphere drops, or GMS drops) represent a novel, topical ocular drug delivery system capable of releasing drug over customizable and sustained periods of time.

Here we present data that suggest IOP reduction in a healthy rabbit model for 28 days following a single administration of the GMS drop loaded with the common glaucoma drug brimonidine tartrate^31^. We also demonstrate retention of this soft, pliable solid hydrogel depot in the inferior conjunctival fornix for the length of the study, which enables drug release well beyond typical retention times for standard eye drops. These IOP lowering results throughout were comparable or superior to twice daily administration of traditional brimonidine eye drops. We also show IOP effects (or lack thereof) in the contralateral control eye as a measure of systemic absorption of drug from the drop versus the depot.

## Results

**Figure 1:**
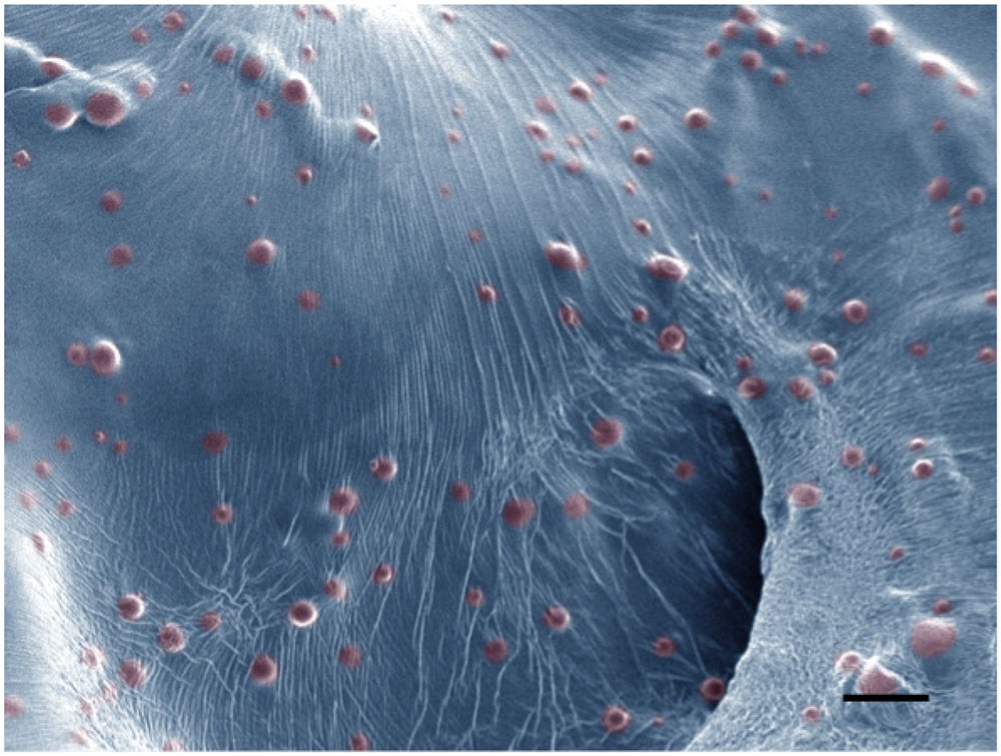
Homogeneous suspension of microspheres in gel matrix. This representative scanning electron microscope (SEM) image of pNIPAAm gel (blue, color added) containing embedded, drug-loaded PLGA microspheres (red, color added) shows that microsphere structure and morphology is maintained and that the microspheres are homogeneously suspended in the gel. Scale bar = 20 μm.

The combined system of microspheres and reverse thermal gel, or GMS drops, was characterized for physical and chemical properties prior to use *in vivo*. The results of an extensive panel of testing for the microspheres alone can be found in a previously published study^21^. Briefly, microspheres had a primarily poreless morphology and a volume average diameter of 7.46 ± 2.86 μm. Upon mixing, the microspheres were confirmed to be incorporated into the pNIPAAM gel matrix via electron microscopy, as seen in the representative image in Fig. 1 (color added to show location of microspheres).

**Figure 2:**
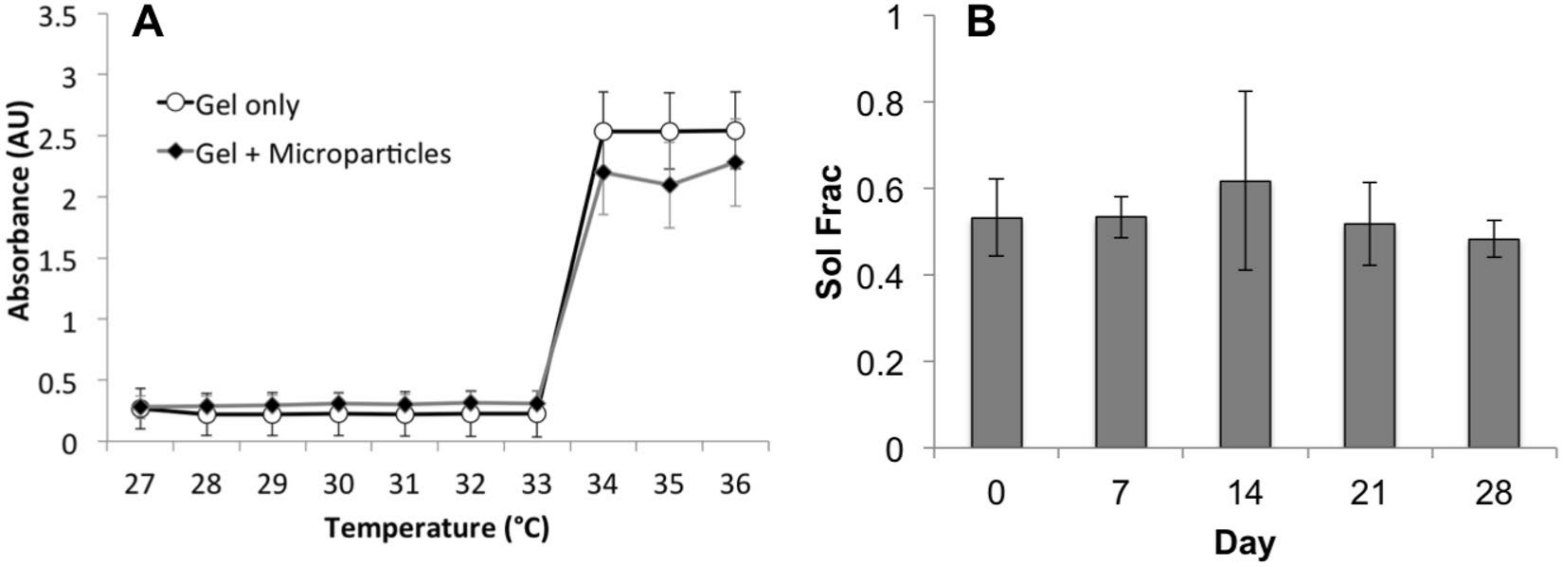
Characterization of gel properties. This includes (**A**) least critical solution temperature (LCST) determination via absorbance at 415 nm vs. temperature measurements for n = 3 gel samples and (**B**) degradation at 37 °C over 28 days, also n = 3. Error bars represent the mean ± standard deviation. Degradation samples were tested for significance at each time point using student’s t test.

The gel itself was additionally characterized for properties relevant to drug release and *in vivo* performance, including LCST, degradation, and swelling ratio. Figure 2A shows absorbance measurements at varying temperatures, indicating an LCST of 33.5 °C. This represents the temperature at which the swollen gel (capable of taking on large volumes of water while remaining insoluble) sheds excess water and shrinks to its solid form. Degradation of gels held at 37 °C for 28 days was negligible, as demonstrated by the solid fraction (Fig. 2B), defined as a ratio of change from initial mass to mass at a given time point. The swelling ratio, a comparison of mass change upon swelling in water, was calculated to be 7.50 ± 0.04 for five gel samples. This value is consistent with other pNIPAAm gels grafted with hydrophilic co-monomers, commonly used as three-dimensional cell scaffolds^32, 33^.

**Figure 3:**
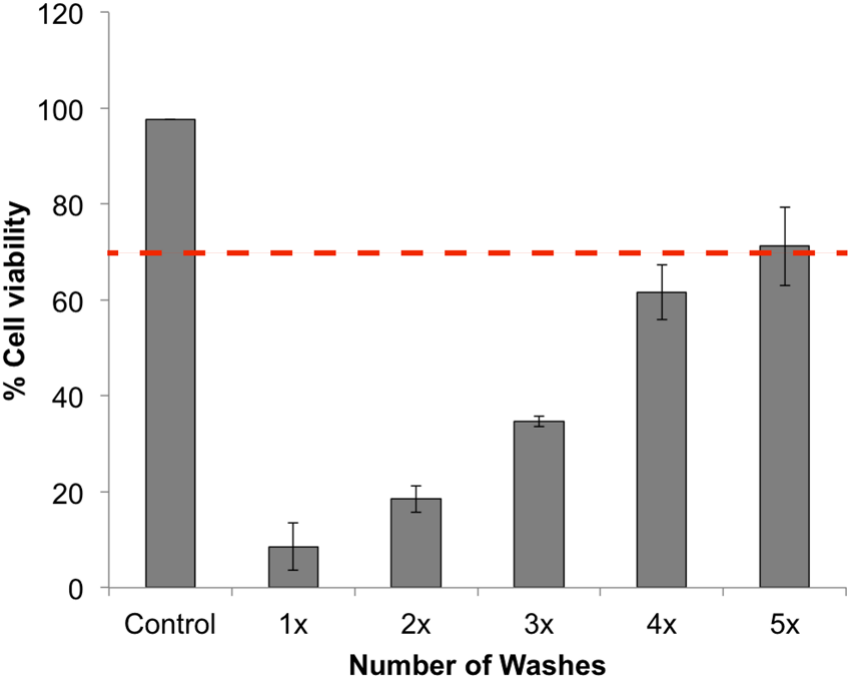
Determining the number of gel washes required to achieve acceptable cell viability. Chang conjunctival cell viability for gels washed 1–5 times (n = 8 for each condition) compared to positive control (no gel). The dashed line represents 70% viability, the minimum threshold for viability recommended for medical devices(35). Error bars represent the mean ± standard deviation.

Conjunctival epithelial cells were chosen as a representative cell line for preliminary cytotoxicity testing. Due to the toxic nature of some of the gel reactants, cell viability was measured for varying numbers of post-synthesis washes to determine the appropriate amount of washing for *in vivo* study preparation. Figure 3 shows the results of n = 8 gel samples for each testing condition. Also shown on Fig. 3 is a line representing 70% viability, the recommended minimum threshold for medical devices such as keratoprostheses^34^ as well as the standard acceptable minimum for ocular toxicity according to the Globally Harmonized System (GHS) classification by the Occupational Safety and Health Administration (OSHA)^35^.

**Figure 4:**
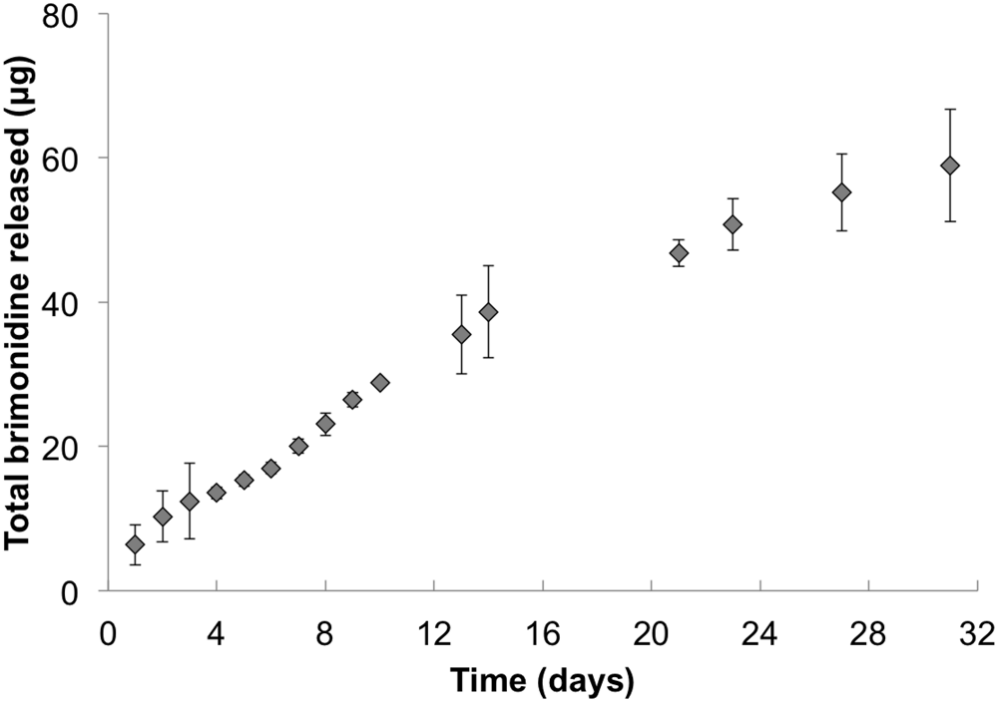
Drug release kinetics from microspheres suspended in hydrogel. *In vitro* brimonidine release from BT-loaded microspheres embedded in pNIPAAm hydrogel (n = 3). Error bars represent the mean ± standard deviation.

As with the microsphere characterization, *in vitro* drug release from the microspheres alone was first demonstrated in our previous study using subconjunctival injection^21^. Similar drug release studies were performed on the gel-based GMS drops containing drug-loaded microspheres to determine if the gel carrier impacted release. Brimonidine concentration over time can be seen in Fig. 4, with the BT release kinetics closely mirroring that of the microspheres alone.

**Figure 5:**
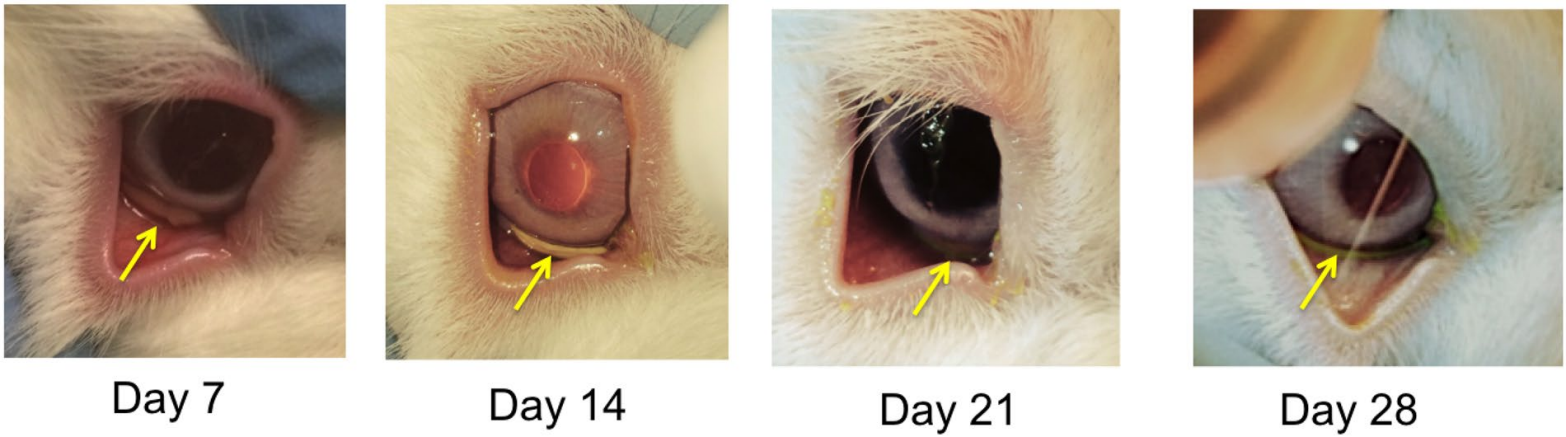
Retention of GMS drops throughout the *in vivo* study. Representative images of gels stained with fluorescein in the inferior fornix over the 28-day study. Arrows indicate the location of the gels, which was confirmed at each time point using cobalt blue light.

The topical GMS drug delivery system was then tested in a healthy rabbit model to confirm hypotensive effects of the drug being released from the depot and to monitor retention in the conjunctival cul de sac over time. Upon administration, all GMS drops in the experimental treatment and negative control groups (n = 5 for each) were confirmed to be present in the lower fornix, with all but one temporally situated. For a more detailed view of drop instillation, please see the Supplementary Video. The location of the GMS drops depot over time can be seen in the representative images in Fig. 5. Complete removal of the GMS drops from the fornix was separately confirmed to be achievable through simple flushing with room temperature saline solution, as seen in the Supplementary Video.

**Figure 6:**
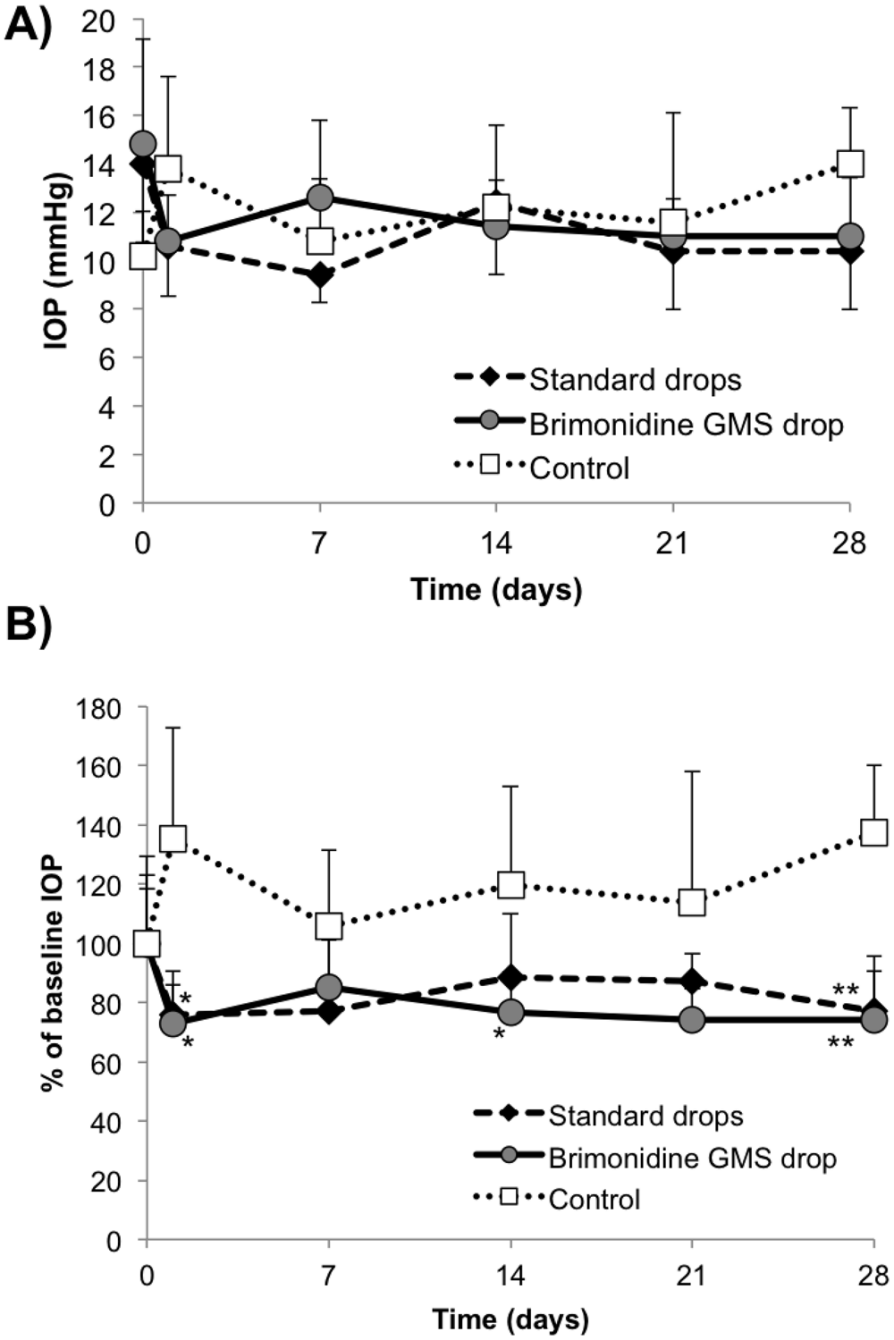
Intraocular pressure (IOP) results in treated eye. Comparison of the *in vivo* hypotensive effect of aqueous BT drops and BT-loaded gel/microsphere drops in the treated eye (OD) as determined by (**A**) actual IOP and (**B**) percent change in IOP from the average baseline value. Gel drops containing no drug served as the negative control. Error bars represent the mean + standard deviation. Statistical significance determined using student’s t-test with *p < 0.05 and **p < 0.01 versus control.

IOP measurements were used as the primary indicator of drug release throughout the study. Prior to administering any treatment, baseline IOP was measured and determined to be not significantly different between treatment groups. Figure 6A and B show the actual IOP measurements and percent change from baseline value in the treated eye, respectively. No statistically significant differences in actual IOP were observed between groups in the treated eye (Fig. 6A). Relative IOP values in Fig. 6B were significantly lower compared to the negative control for both the BT and gel drops on days 1 (p < 0.05) and 28 (p < 0.01). Additionally, relative IOP was significantly lower in the gel drop animals on day 14 compared to the negative control, with p < 0.05.

**Figure 7:**
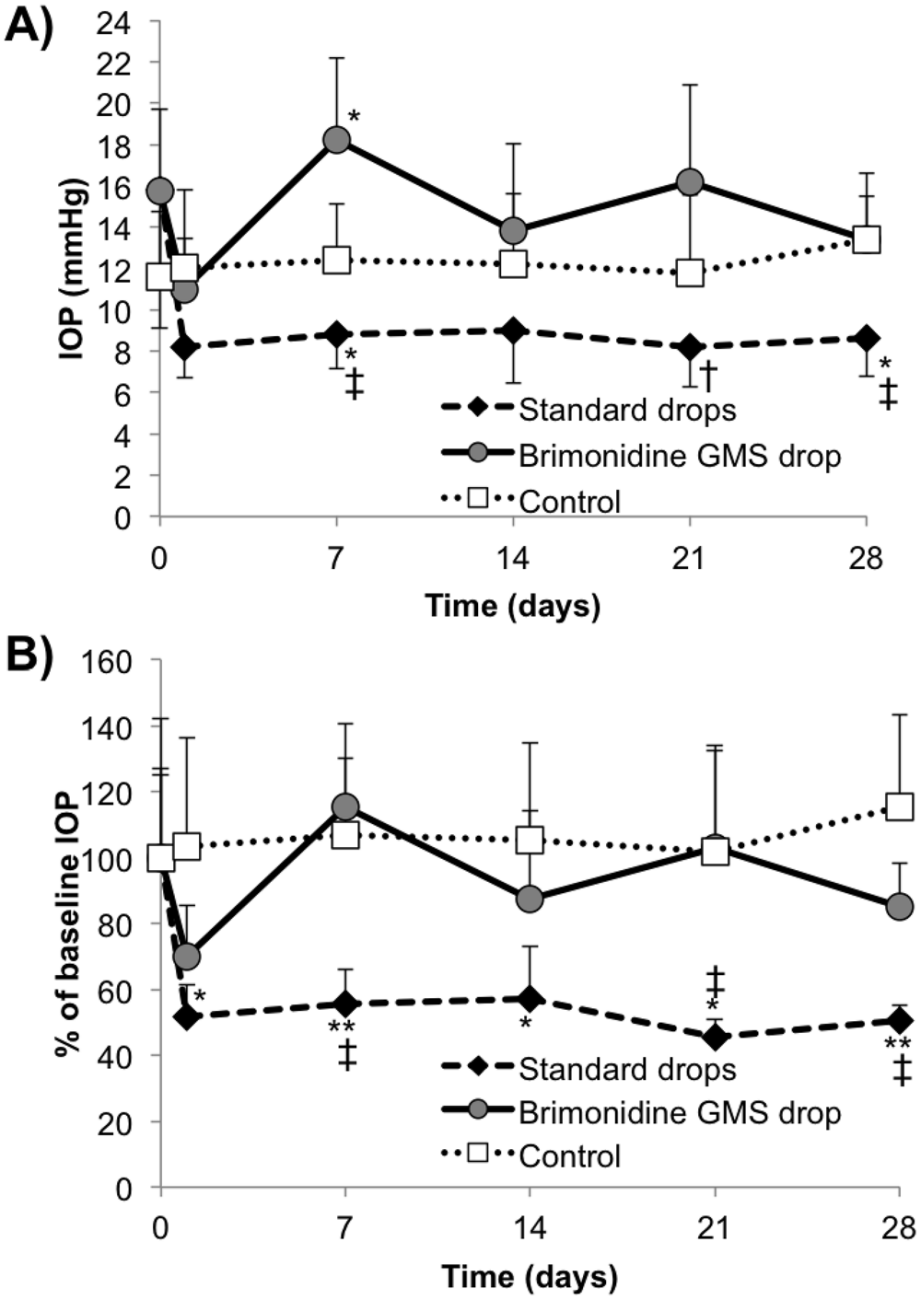
Intraocular pressure (IOP) results in untreated (contralateral) eye. Comparison of the *in vivo* hypotensive effect of aqueous BT drops and BT-loaded gel/microsphere drops in the untreated, contralateral eye (OS) as determined by (**A**) actual IOP and (**B**) percent change in IOP from the average baseline value. Gel drops containing no drug served as the negative control. Error bars represent the mean + standard deviation. Statistical significance determined using student’s t-test with *p < 0.05 and **p < 0.01 versus control and ^†^p < 0.05 and ^‡^p < 0.005 versus gel drops.

The same metrics can be seen for the contralateral, untreated eye in Fig. 7A and B. Aqueous BT drops resulted in significantly lower IOP compared to the negative control on days 7 and 28 (p < 0.05), as seen in Fig. 6A. The BT drops also demonstrated significantly lower actual IOP values compared to the gel drops, with p < 0.005 on days 7 and 28 and p < 0.05 on day 21. Relative IOP percentages in Fig. 6B can be considered significantly lower in the BT group, with *p* < 0.005 versus gel drops on days 7, 21, and 28. Animals receiving BT drops also showed significantly greater decreases relative to baseline compared to the negative control, with p < 0.05 on days 1, 14, and 21 and p < 0.01 on days 7 and 28.

## Discussion

Adherence to medical therapy is one of the greatest barriers to effective management of glaucoma. The overwhelming majority of glaucoma patients are non-adherent to their treatment regime in some way, putting them at much higher risk for vision loss^36^. Non-adherence may be intentional (failure to fill prescription^37^ or administer drops^5^) or unintentional (failure to instill drops correctly^9^).

Topical administration offers the potential to provide a high degree of safety compared to subconjunctival or intraocular injection; however, controlled release technology for glaucoma has yet to be successfully translated to a fully non-invasive and long-term formulation. Although nanoscale eye drop additives have demonstrated enhanced biocompatibility and bioavailability compared to topical drops alone^38–40^, drug release can only be sustained for several days or less. Other experimental formulations, such as drug-loaded ocular inserts that can release drug for one month or more^41–43^, require patients to learn new methods of self-administration that can be cumbersome, particularly for elderly patients. Such inserts do not address the issue of patient discomfort, which has been previously reported^44, 45^. To date, there still remains a need for an easy to instill, topically administered formulation that can sustain the release of drugs over extended periods of time.

Ophthalmic drug delivery systems currently in development can generally be classified into one of two categories: (1) hours to days of drug release from a patient-administered device or drop or (2) weeks to months of drug release from a clinician-administered device^16^. In contrast, we have developed a four-week, topical treatment option for glaucoma designed for easy, self-administration by patients. Inclusion of a hydrogel matrix expands upon our previously published work using controlled release to deliver glaucoma drugs in a non-topical administration format (subconjunctival^21^). The specific gel properties were chosen according to their clinical applicability from various literature reports of hydrogel fabrication and resulting properties, as discussed further below.

This new delivery system consists of drug-loaded microparticles that provide highly-tunable, long-term release suspended in a thermoresponsive hydrogel eye drop, resulting in an easy to instill, comfortable format for long-term topical retention of the formulation. Unlike many long-term glaucoma drug delivery systems in development, the retaining gel is non-degradable and designed to be removed when instilling a new dose. Further, the lyophilized microsphere formulation requires only simple mixing just prior to instillation and eliminates the need for preservatives. This gel microsphere eye drop platform (GMS drops) may offer a drastic decrease in dosing frequency without sacrificing hypotensive efficacy, as suggested by our initial *in vivo* results. The system described herein also obviates the need for more invasive administration methods, which can result in IOP spikes and other adverse events^26, 27^. Importantly, the drops were easily administered by non-clinical personnel with no sedation of animals whatsoever.

The primary measure of efficacy for glaucoma drug delivery is a decrease in IOP. Our results suggest that, according to that metric, the gel-based drops are comparable to aqueous drops. Both treated groups resulted in significantly lower IOP relative to the baseline value when compared to control animals receiving no drug at various time points throughout the study. Although baseline IOP measurements between groups were not significantly different, the data were normalized for comparison because of the noticeably lower baseline value in the negative control group. This may have also contributed to the relative IOP increase in the negative control group, an observation which warrants further investigation in future studies. The use of normotensive rabbits results in a moderate decrease in IOP, which is not uncommon^46–48^ and actually provides a more rigorous model for determining significance of our experimental treatment. In the future, these studies will be extended to a rabbit glaucoma model using a larger number of animals per group, as others have done previously^49–51^, to better demonstrate the *in vivo* efficacy of our treatment method. We hypothesize that, as others have shown, the effect of our treatment will be magnified when baseline pressures are higher. Regardless, the ease of administration and statistically significant effects on IOP are a necessary and promising step for clinical translation of the gel drops.

The phenomenon of asymmetric bilateral IOP reduction (i.e. a more pronounced effect in the untreated eye) has been observed previously in topical administration of α_2_ adrenoceptor agonists in both rabbits^52^ and humans^53–56^. While this effect was noted in the positive control arm, IOP measurements in the untreated, contralateral eye suggest significantly lower systemic absorption of drug, similarly to our previous study with subconjunctival injection^21^. We hypothesize this is due to the greatly decreased concentration of drug used in the controlled release depot compared to traditional drops. To test this hypothesis, future studies will examine drug concentration in each eye and in plasma at time points throughout the study. As our *in vitro* data does not suggest that there is any diffusion hindrance of drug exiting the polymer microspheres due to the gel matrix, we would anticipate these drug levels would be the similar to those measured in the subconjunctival depot of microspheres alone.

We observed no evidence of inflammation or irritation, suggesting biocompatibility of the materials in the fornix. This agrees well with our aforementioned preliminary results, which suggests a minimum of 5 washing steps for use of gels *in vivo*. Although our studies show no subsequent negative side effects, future investigation will be performed via histological examination at each time point throughout the study. For example, degradation of the PLGA microspheres has been demonstrated previously to result in a slight pH decrease in the local microenvironment^57–59^. We do not anticipate any issues due to the small quantity of microspheres being used and the lack of direct contact with the ocular surface resulting from the hydrogel carrier. Similarly, although the depot is situated in the fornix, we will explore any potential involvement of the cornea due to the highly sensitive nature of that tissue.

Removal of our rabbits’ nictitating membrane was necessary for long-term retention of the gel drops, and more closely approximates the human fornix, resulting in 100% retention of the drops for the full length of the study. As with the gel drops themselves, we similarly did not see any sign of lingering inflammation or effect on IOP due to resection of this tissue in rabbits receiving the gel drops. The pliability and low volume of the gel drops, as well as their opacity which makes them readily visible, will likely make this drug delivery system well-tolerated and retained in human eyes. Additionally, several studies modeling the temperature of the fornix relative to ambient temperature suggest that significant drops in temperature would not lead to phase transition of the gel drop back to a liquid^60, 61^. Indeed, these properties were engineered into the final gel based on previous reports of pNIPAAm-based hydrogels. In particular, the lack of additional monomer prevents significant change from the pNIPAAm LCST of 33 °C^62–64^, the inclusion of PEG as a grafting molecule creates a “softer” gel that remains intact^32^, lower weight percent of monomer with high PEG content forms an opaque gel^32^, and exclusion of additional monomer along with addition of PEG maintains low viscosity^32, 62, 63, 65^.

The rapid liquid-gel transition, pliability, opacity, and drug release properties of this unique ophthalmic drug delivery system make it an attractive candidate for treatment of glaucoma. To our knowledge, this represents the first long-term drug releasing depot that can be administered as simply as a traditional eye drop. Our results suggest that IOP reduction with a single GMS drop is comparable to that of twice-daily standard drops with a significant decrease in systemic absorption. Further, the GMS drops are easily retained and well tolerated. As we move toward translation of the GMS drops, we will explore pharmacokinetics of the released drug in a glaucoma model.

## Methods

All materials and reagents were obtained from Sigma Aldrich (St. Louis, MO) unless otherwise specified.

### Microsphere fabrication and characterization

Brimonidine tartrate (BT)-loaded poly(lactic-co-glycolic) acid microspheres were fabricated as described previously, using a double emulsion procedure^21^. Briefly, 200 mg of PLGA (MW 24–38 kDa, viscosity 0.32–0.44 dl/g) was dissolved in 4 ml of dicholoromethane (DCM). To this solution, 250 μl of a 50 mg/ml aqueous BT solution was added (prepared from solid BT, Santa Cruz Biotechnologies, Santa Cruz, CA). This suspension was then sonicated for 10 s and homogenized for 1 min in 2% poly(vinyl alcohol) (Polysciences) at 7000 rpm (Silverson L4RT-A). The emulsion was then mixed with a 1% PVA solution for 3 h to allow residual DCM to evaporate. The microspheres were then washed via centrifugation with deionized (DI) water prior to lyophilization for 48 hours (Virtis Benchtop K Freeze Dryer, Gardiner, NY). Dry microspheres were stored at −20 °C until use.

Drug-loaded microspheres were characterized for average size and surface morphology using volume impedance measurements (Multisizer 3 Coulter Counter, Beckman Coulter, Indianapolis, IN) and scanning electron microscopy (SEM, JEOL 6335 F Field Emission SEM, Peabody, MA). The volume average microsphere diameter was determined for a minimum of 10,000 microspheres. SEM images were also obtained for gel samples containing the microparticles (combined via passive mixing, as described below).

### Gel fabrication and characterization

The choice of materials and conditions for gel formation was informed by the extensive body of literature regarding their effects on physical and chemical properties (see Discussion section). Reverse thermal hydrogels were prepared via aqueous free radical polymerization by adding 0.1 ml poly(ethylene glycol) (MW 200 Da) to 2.0 ml DI water. To this solution, 0.1 g of n-isopropylacrylamide (NIPAAm) was added and vortexed until the solution was homogeneously mixed. Polymerization was achieved by adding 30 μl of ammonium persulfate (APS, 0.1 mg/ml in DI water) and 5 μl of tetramethylethylenediamine (TEMED) as the redox-pair initiators and refrigerating overnight. The resulting gel, approximately 1 ml as described, was washed five times in DI water heated to 50 °C prior to incorporation of PLGA microspheres.

The lower critical solution temperature (LCST), the temperature at which deswelling and gelation occur, was determined using absorbance measurements on a plate reader for n = 3 gel samples. Temperature was increased by 1° increments and absorbance was determined at a wavelength of 415 nm (SpectraMax M5, Molecular Devices, Sunnydale, CA). These measurements were repeated for gels alone and combined with the PLGA microspheres. The absorbance values were also tested for repeatability in the reverse conditions by cooling rather than heating the gels.

The degradation rate of the gels was determined by measuring the solid fraction remaining after varying amounts of time, as reported by others^66^, according to the following equation:

1 $$Sol\,Frac\,( \% )=\frac{{M}_{i}-{M}_{d}}{{M}_{d}}$$

where M_i_ is the initial mass of the gel after crosslinking and M_d_ is the mass of the dry gel after swelling. Briefly, identical gel samples were massed and incubated for the appropriate amount of time at 37 °C prior to determining percent loss, if any. Degradation at each time point was calculated as the average of three samples ± standard deviation and measurements were taken on days 0, 7, 14, 21, and 28.

Swelling ratio was determined as the average of five samples ± standard deviation according to the following relationship:

2 $$Swelling\,Ratio=\frac{{M}_{s}-{M}_{d}}{{M}_{d}}$$

where M_s_ represents the mass of the gel after swelling in sufficient volumes of PBS. Gel samples were dried at 37 °C for approximately 72 hours prior to recording individual M_d_ values.

Cytotoxicity of the gels was determined by incubating gel samples with Chang conjunctival epithelial cells (ATCC). Briefly, 100 μl aliquots of 10^5^ cells/ml in growth medium were added to wells in a 96-well plate. These were expanded to achieve a monolayer of cells, after approximately 2–3 days. Cells with medium only were used as the positive control for viability. Polymerized gel samples that had undergone 1–5 washing cycles were added to wells in 100 μl aliquots along with 100 μl of growth medium. These samples were incubated at 37 °C with 5% CO_2_ for 24 h followed by addition of 20 μl of PrestoBlue® viability agent (Life Sciences) and additional incubation for a total of 20 min. Triton X100 was added to negative control wells as a lysis agent and incubated for a total of 15 min. Fluorescence was then determined in each well using an emission filter of 500 nm and an excitation filter of 620 nm. The mean and standard deviation absorbance values were determined for n = 8 samples of each test group. Percent viability was determined as follows:

3 $$ \% \,Viability=100\times [1-\frac{({F}_{PositiveControl}-{F}_{Gelsample})}{({F}_{PositiveControl}-{F}_{LysisControl})}]$$

where F is an average fluorescence value and each gel sample represents a different number of washing cycles.

### *In vitro* drug release

*In vitro* drug release studies were performed to assess the kinetics of brimonidine release from the microspheres, as described in our previous study^21^. Known masses of lyophilized microspheres were suspended in phosphate buffered saline (PBS) and incubated at 37 °C. These samples were centrifuged for 5 min at 1000 rpm and the supernatant removed for analysis of brimonidine content. The supernatant was then replaced with fresh PBS and samples were vortexed briefly prior to additional incubation. The same procedure was repeated for microspheres embedded in the hydrogel matrix, with the exception that samples were not centrifuged prior to removing and replacing the supernatant. All gel samples were prepared by suspending microspheres at a maximum of 1 mg/10 μl gel, previously determined to be the optimal concentration for pipetting. Brimonidine concentration in supernatant samples was determined via UV/Vis absorption using a microplate reader set to a wavelength of 320 nm. Background signal from microspheres containing no drug was subtracted prior to reporting results as the average of n = 3 samples for each test condition (microspheres alone and microspheres in gel) ± standard deviation.

### *In vivo* studies

The protocols for performing all animal studies were reviewed and approved by Institutional Animal Care and Use Committee (IACUC) of the University of Pittsburgh and all studies were conducting according to the Association for Research in Vision and Ophthalmology (ARVO) Statement for Use of Animals in Ophthalmic and Vision Research.

*In vivo* efficacy testing of the GMS drops for glaucoma was performed on healthy New Zealand white rabbits over 28 days. Five animals per group were randomized to one of the following groups: twice daily 0.2% BT drops, single BT-loaded GMS drops, and single administration of the GMS drops containing drug-free blank microspheres. Prior to beginning the study, the ten rabbits receiving the gel drops underwent resection of the nictitating membrane in their right eye only in order to facilitate GMS drop retention. This procedure was minimally invasive and required topical anesthetic only. As detailed below, the study did not begin until IOP measurements had returned to their baseline values and there was no evidence of any lingering inflammation (approximately one week).

All IOP measurements were taken by the same technician using the TonoVet rebound tonometer (Icare, Finland) between 9–9:30 am, each time beginning with the left eye. Prior to resection of the nictitating membrane, four individual baseline measurements were recorded per animal on consecutive days. Rabbits had been randomized to a treatment group prior to beginning IOP measurements. Following the resection, these measurements were repeated for three consecutive days to confirm that IOP levels had returned to their baseline values.

On Day 0 of the study, five animals were randomly assigned to receive a single drop comprised of BT-loaded or blank (drug-free) microspheres embedded in hydrogel. Hydrogel and microsphere materials were stored separately until use to prevent premature drug release. The 100 μl drop containing 10 mg of drug-loaded microspheres was administered via eye dropper to awake, non-restrained, non-sedated rabbits in the right eye only (see video of drop administration in Supplemental Information). The drop was administered as deep in the lower fornix as possible and visually confirmed to be temporally situated following gelation. Rabbits in the negative control group received a single administration of gel with blank microspheres containing no drug. Rabbits in the positive control group received a single drop of BT solution in the right eye only twice daily for the entire length of the study, between 8–8:30 am and again between 5–5:30 pm. IOP measurements were taken on both eyes throughout the study as described previously, always within a minimum of 30 minutes and a maximum of 60 minutes after instilling the morning BT drop. The left eye remained untreated in all rabbits throughout the study.

Eyes were regularly checked for signs of infection or inflammation by a masked observer by instilling sodium fluorescein drops and examining with a portable slit lamp containing a cobalt blue light (Reichert Technologies, Depew, NY). Gels were visually located in the lower fornix at each post-administration time point - days 1, 7, 14, 21, and 28 of the study - via fluorescein staining and visualization under cobalt blue light.

### Statistics

One-way analysis of variance (ANOVA) was performed on baseline IOP measurements to determine statistically significant differences, if any, in starting IOP values for subsequent comparisons. The same methods were used to confirm that IOP had returned to baselines values following resection of the nictitating membrane. We then calculated the percent change in IOP at each time point relative to the average baseline IOP for each group. The Student’s *t*-test was used with a two-tailed distribution to compare actual and differential IOP data from the aqueous and gel drop groups. Statistically significant differences were designated by a significance criterion (*p* value) below 0.05.

## Electronic supplementary material

Video of gel instillation and removal

Video legend
