# Supplementary material for: Long Term Glaucoma Drug Delivery Using a Topically Retained Gel/Microsphere Eye Drop
Source: Sci Rep. 2017 Aug 17;7:8639. doi: 10.1038/s41598-017-09379-8 (PMC5561248; doi:10.1038/s41598-017-09379-8)
Supplement: Supplementary file 2 — Video legend [file 41598_2017_9379_MOESM2_ESM.pdf]

Long Term Glaucoma Drug Delivery Using a Topically Retained Gel/Microsphere Eye Drop

Morgan V. Fedorchak, PhD[1,2,3,4,7], Ian P. Conner, MD, PhD[1,2,7], Joel S. Schuman, MD[1,2,4,5,7-11], Anthony Cugini[5], Steven R. Little, PhD[1,3,5,6,7]

[1]UPMC Eye Center, Department of Ophthalmology, University of Pittsburgh School of Medicine; [2]The Louis J. Fox Center for Vision Restoration; University of Pittsburgh Departments of [3]Chemical Engineering, [4]Clinical and Translational Science, [5]Bioengineering, and [6]Immunology; [7]The McGowan Institute for Regenerative Medicine; NYU Langone Eye Center, Departments of [8]Ophthalmology, [9]Neuroscience and [10]Physiology, NYU School of Medicine; [11]Depatment of Electrical and Computer Engineering, NYU Tandon School of Engineering.

Supplemental Information

Video demonstrating instillation (via eye dropper) and removal (by flushing with room temperature saline) of BT-loaded gel/microsphere eye drop in the inferior fornix of New Zealand white rabbit. Still images demonstrate the placement of the drop and subsequently, that no gel fragments remained upon removal, as determined by staining with fluorescein and visualization with cobalt blue light.
